# Supplementary material for: Impact of Enniatin B and Beauvericin on Lysosomal Cathepsin B Secretion and Apoptosis Induction
Source: Int J Mol Sci. 2023 Jan 19;24(3):2030. doi: 10.3390/ijms24032030 (PMC9916760; doi:10.3390/ijms24032030)
Supplement: Supplementary file 1 [file ijms-24-02030-s001.zip › ijms-2094404-supplementary.pdf]

Supplementary Data

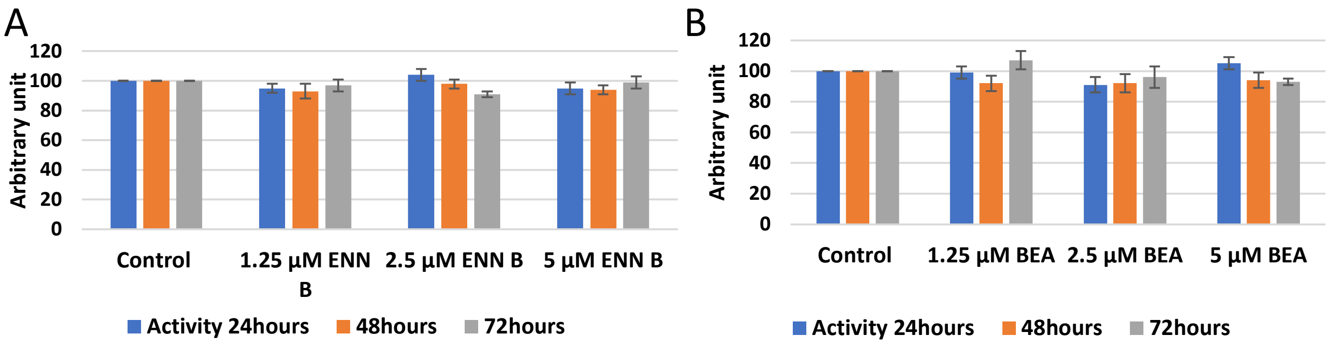

Figure S1. Impact of ENN B and BEA on proteolytic activities of cathepsin L in cellular extracts. (**A**) Cathepsin L activities were measured using cathepsin L specific substrate Z-F-R.AMC in cellular extracts after treatment with different ENN B and different periods concentrations. (**B**) Cathepsin L activities measured after treatments with different concentration for different periods with BEA. The data were analyzed using two-way ANOVA with Bonferroni's or Dunnett's post hoc analysis (**p* < 0.05; ***p* < 0.01; ****p* < 0.001; N = 7). Statistics was calculated using GraphPad Prism.

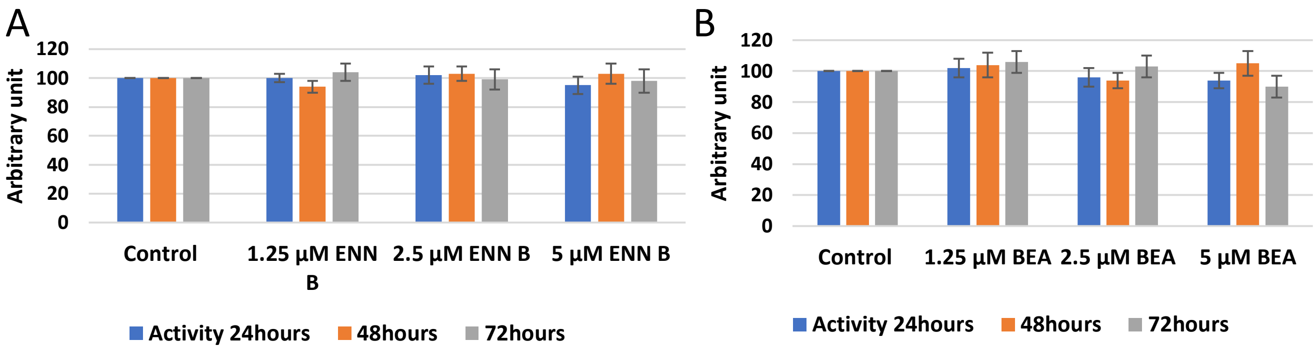

Figure S2. Impact of ENN B and BEA on proteolytic activities of Cathepsin L in media. (**A**) cathepsin L activities were measured using cathepsin L specific substrate Z-F-R.AMC in media after treatment with different ENN B and different concentrations for different periods. (**B**) cathepsin L activities measured in media after treatment with different concentrations of BEA for different periods. The data were analyzed using two-way ANOVA, Statistics was calculated using GraphPad Prism.

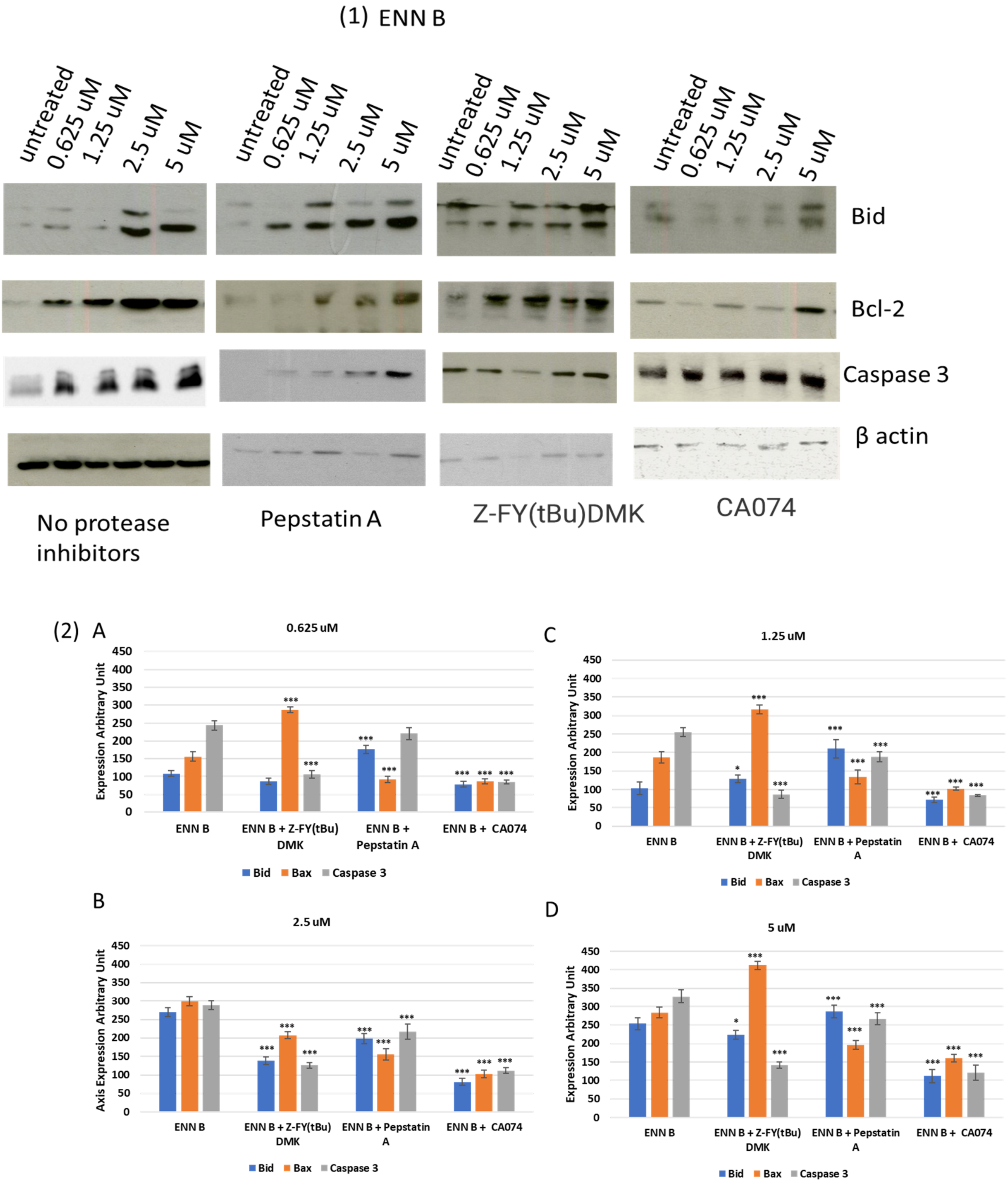

Figure S3. Impact of ENN B treatment on the expressions of some apoptotic proteins. (3.1) Western blot analysis of cleaved Bid, cleaved Bcl-2 and cleaved caspase 3 expression in KB-31- cells treated with 0, 0.625, 1.25, 2.5 and 5 μM ENN B for 24 h in the presence and absence of 10 μM of cathepsin D inhibitor pepstatin A or the same concentration of cathepsin L inhibitor Z-FY (tBu)DMK or cathepsin B inhibitor CA074. β-actin was used as an internal control. (3.2, **A**, **B**, **C** and **D**) Evaluation of cleaved Bid, cleaved Bcl-2 and cleaved caspase 3 expression after treatment with 0, 0.625, 1.25, 2.5 and 5 μM ENN B for 24 h in the presence and absence of 10 μM of cathepsin D, cathepsin L or cathepsin B specific inhibitors. The data were analyzed using two-way ANOVA with Dunnett's post hoc analysis (*$p < 0.05$; **$p < 0.01$; ***$p < 0.001$; N = 7). Statistics was calculated using GraphPad Prism.

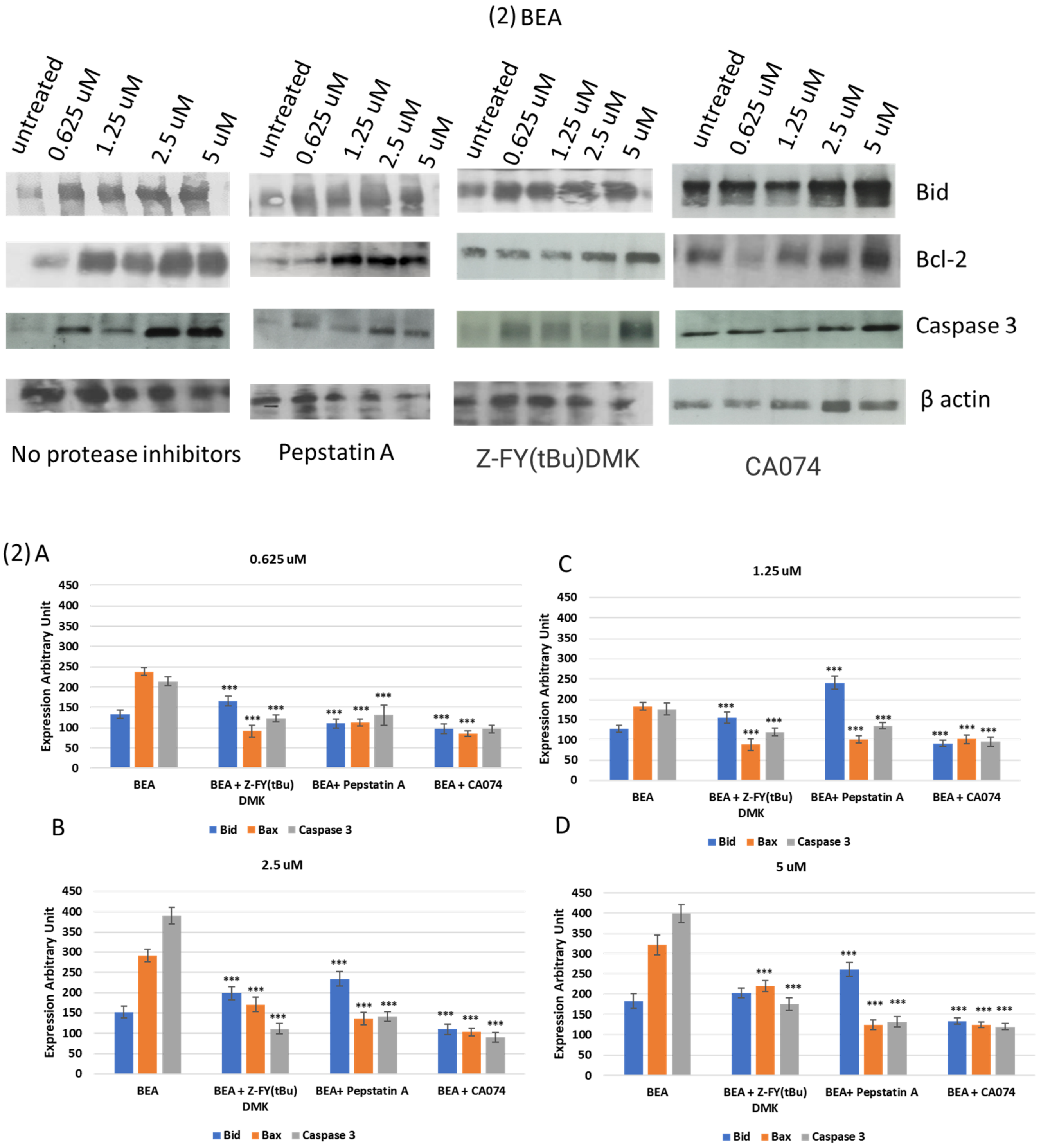

Figure S4. Impact of BEA treatment on the expressions of some apoptotic proteins. (4.1) Western blot analysis of cleaved Bid, cleaved Bcl-2 and cleaved caspase 3 expression in KB-31- cells treated with 0, 0.625, 1.25, 2.5 and 5 µM BEA for 24 h in the presence and absence of 10 µM of cathepsin D inhibitor pepstatin A or the same concentration of cathepsin L inhibitor Z-FY (tBu)DMK or cathepsin B inhibitor CA074. β-actin was used as an internal control. (4.2, **A**, **B**, **C** and **D**). Evaluation of cleaved Bid, cleaved Bcl-2 and cleaved caspase 3 expression after treatment with 0, 0.625, 1.25, 2.5 and 5 µM BEA for 24 h in the presence and absence of 10 µM of cathepsin D, cathepsin L or cathepsin B specific inhibitors. The data were analyzed using two-way ANOVA with Dunnett's post hoc analysis (*$p < .05$; **$p < .01$; ***$p < .001$; N = 7). Statistics was calculated using GraphPad Prism.
